# Therapist-supported versus self-guided digital CBT for chronic pain with neurodevelopmental traits: A pilot feasibility randomized controlled trial

**DOI:** 10.1016/j.invent.2026.101020

**Published:** 2026-11-04

**Authors:** Kayoko Taguchi, Mirai Miyoshi, Rieko Takanashi, Emiko Oshita, Miyuki Furukawa, Yoshiyuki Hirano, Eiji Shimizu

**Affiliations:** a Research Center for Child Mental Development, Chiba University, Chiba, Japan; b School of Health Sciences, Faculty of Medicine, University of the Ryukyus, Okinawa, Japan; c Faculty of Liberal Arts, The Open University of Japan, Chiba, Japan; d United Graduate School of Child Development, The University of Osaka, Osaka, Japan; e Department of Cognitive Behavioral Physiology, Graduate School of Medicine, Chiba University, Chiba, Japan

**Keywords:** Chronic pain, Neurodevelopmental traits, Cognitive behavioral therapy, Guided digital CBT, Self-help, Pilot randomized controlled trial

## Abstract

**Background:**

Chronic pain frequently co-occurs with neurodevelopmental disorders (NDDs) such as autism spectrum disorder (ASD) and attention-deficit/hyperactivity disorder (ADHD). If these traits are overlooked, standard cognitive behavioral therapy (CBT) may be less effective. We developed a brief digital CBT program that adds psychoeducation on NDD traits to standard CBT for chronic pain. This study primarily examined its feasibility in adults with chronic pain and comorbid NDDs.

**Methods:**

In this three-arm pilot feasibility randomized controlled trial, 44 adults with chronic pain and a self-reported diagnosis of ASD or ADHD were randomized to therapist-supported digital CBT (n = 15), self-guided digital CBT (n = 16), or a sham condition (n = 13; daily submission of the weekday and weather). The program comprised ten 15-min videos over two weeks. Primary outcomes were feasibility and pain catastrophizing; depressive symptoms were exploratory. Mixed models for repeated measures were used.

**Results:**

Post-intervention retention was 90.9% (40/44). Of 31 participants allocated to CBT, 29 (93.5%) completed the program, all rating it as safe. Compared with sham, pain catastrophizing decreased significantly more in both the self-guided and therapist-supported groups (*p* = .006 and *p* = .035), with no clear difference between the two CBT formats. Depressive symptoms showed a similar pattern. Other outcomes did not differ.

**Conclusions:**

A brief digital CBT program for this population appears feasible and acceptable. Preliminary estimates favored both CBT formats but were imprecise because the trial was not powered for between-group comparisons. An adequately powered trial with follow-up and systematic assessment of negative effects is needed.

## Highlights

• Brief digital CBT was feasible in adults with chronic pain and NDD traits.
• Retention was 90.9%; 96.7% of those who started completed the program.
• Nearly all participants viewed every video, but homework practice was modest.
• Pain catastrophizing fell more with both CBT formats than sham; estimates imprecise.
• No difference was found between therapist-supported and self-guided formats.

## 1 Introduction

Psychological treatments such as CBT yield only modest benefits for many patients with chronic pain (Williams et al., 2020). Increasing evidence indicates that neurodevelopmental disorders (NDDs), such as ASD and ADHD, frequently co-occur with chronic pain (Garriga-Cazorla et al., 2025; Mundal et al., 2024). Both ASD and ADHD are classified as NDDs in the DSM-5 (American Psychiatric Association, 2013). Our previous study also showed that ASD and ADHD traits were common among patients with somatic symptom disorder, including chronic pain; specifically, chronic pain was associated with difficulty identifying feelings, a feature linked to ASD traits, and with impulsivity and extraversion, features related to ADHD (Miyoshi et al., 2025). If these traits are overlooked or underestimated, standard treatment for chronic pain may be less effective. Such traits are easily overlooked: ADHD frequently remains undiagnosed in adults, although ADHD traits may be relevant in a subset of patients with chronic pain (Kasahara et al., 2026), and women with ASD are often not diagnosed until adulthood (Lockwood Estrin et al., 2021). A qualitative study in the United Kingdom reported that adolescents with chronic pain and autistic traits experienced pain more intensely because of heightened anxiety, atypical sensory processing and other neurodivergent characteristics, which often limited the benefit of standard treatments (Jordan et al., 2024). Difficulties in emotion regulation and sustained attention, as well as atypical interoception, may also delay the recognition of pain and emotional states (Kasahara et al., 2026). In a study of standard CBT for adults with chronic low back pain, patients who did not respond had higher ADHD traits than those who responded (Shimizu et al., 2021). Beyond chronic pain, a co-occurring diagnosis of ASD or ADHD predicted poorer outcomes of a digital treatment for social anxiety disorder (Hlynsson et al., 2026). In contrast, among adolescents with chronic pain, autistic traits did not reduce the degree of improvement after a highly intensive (more than 80 h) multidisciplinary residential program (Moore et al., 2025). However, that program combined physiotherapy, psychological therapy and parent sessions and did not examine CBT alone; it therefore remains unclear whether brief, low-intensity programs can be effective without considering NDD traits. Moreover, most of this evidence comes from observational, cross-sectional or qualitative studies, and no trial has directly tested whether NDD traits moderate the effects of CBT for chronic pain. Although these findings suggest a need for interventions tailored to NDD traits, few studies have developed concrete interventions for patients with chronic pain and these traits.

Remotely delivered CBT for chronic pain has small benefits on pain intensity (moderate certainty) and functional disability (moderate to low certainty) in adults (Rosser et al., 2023). A recent three-arm randomized controlled trial (RCT) compared health coach-led telehealth CBT, a self-completed CBT program and usual care; both intervention groups showed greater improvements in pain intensity and pain-related disability than usual care, with the largest effect for the health coach-led program (DeBar et al., 2025). However, intervention studies for chronic pain in people with co-occurring NDDs are scarce and have been limited to children and adolescents (Han et al., 2024; Kasahara et al., 2026); to our knowledge, no study has focused on adults. We therefore developed a brief digital CBT program that adds psychoeducation on NDD traits and their relationship to pain to standard CBT components for chronic pain. The aim of this pilot study was to examine the feasibility and acceptability of this program, delivered in therapist-supported and self-guided formats, and to obtain preliminary estimates of change in pain catastrophizing to inform a future definitive trial.

## 2 Methods

### 2.1 Procedure

This study was a three-arm randomized controlled pilot trial conducted at the Cognitive Behavioral Therapy Center, Chiba University Hospital, between October 2024 and March 2025. Participants were recruited over a two-week period in October 2024 from an online research panel managed by NTT Com Online Marketing Solutions Corporation (Tokyo, Japan). Eligible participants were adults aged ≥18 years with chronic pain who met the screening criteria for somatic symptom disorder with predominant pain and reported a diagnosis of ASD or ADHD and who were able to receive the CBT program via LINE, a smartphone-based messaging application (LY Corporation, Tokyo, Japan). Exclusion criteria were (1) severe psychiatric disorders, including neurocognitive disorders, psychotic disorders, bipolar disorders and substance-related disorders (DSM-5); (2) cancer pain; (3) serious progressive physical illness likely to require discontinuation of the study; and (4) involvement of litigation or compensation related to the pain. After providing online informed consent, participants completed a screening assessment comprising: (A) confirmation that pain interfered with daily functioning; (B) assessment of somatic symptom disorder features using the Somatic Symptom Disorder–B Criteria Scale (SSD-12); and (C) current pain intensity and a self-reported diagnosis of ASD or ADHD, assessed with a single yes/no item. No medical records were reviewed, and no structured diagnostic interview was conducted; the item did not distinguish between the two diagnoses. Participants who met criterion A, had an SSD-12 score ≥ 16, a NRS score > 5, and a self-reported diagnosis of ASD or ADHD were enrolled, then, randomized in a 1:1:1 ratio to one of three groups: (1) guided digital CBT with therapist follow-up (CBT-Th.F), (2) self-guided digital CBT without therapist support (CBT-Self), or (3) a sham control condition. Randomization was stratified by age and baseline pain intensity, and allocation was automatically concealed using a computer-generated system. Assessments were conducted at baseline (T0) and immediately post-intervention (T1, Week 2). No follow-up assessments were planned in this pilot trial. Due to the nature of the digital behavioral intervention, participants and intervention providers were not blinded to group allocation. All outcomes were collected using self-report questionnaires administered via Google Forms; therefore, assessor blinding was not applicable. Adherence was monitored by recording video completion and homework submission rates.

### 2.2 Interventions

The experimental intervention program consisted of ten sessions, each comprising a 15-min short video. Short videos delivered once a day were chosen to reduce cognitive load and to accommodate attentional and executive function difficulties, which can hinder adherence to self-guided internet interventions in adults with ADHD or ASD (Nordby et al., 2022; Westerberg et al., 2023), and the two-week format was chosen to keep the burden low and to test feasibility before a longer program. The program combined core components of CBT for chronic pain used in our previous trial (Taguchi et al., 2021) with components targeting NDD-related difficulties identified in our cross-sectional study (Miyoshi et al., 2025) (Table 1). Several videos included a brief guided exercise (e.g., relaxation or attention shifting), and the corresponding homework asked participants to continue practicing the exercise in daily life throughout the remaining program period. Similar components have been included in psychological interventions for neurodivergent adults: psychoeducation about ADHD, attention training, impulse control and emotion regulation in internet-delivered CBT for adults with ADHD (Nasri et al., 2023), and relaxation, cognitive restructuring with behavioral experiments and attention training in a modified CBT program for autistic young adults (Bemmer et al., 2021).

**Table 1:** Overview of the digital cognitive behavioral therapy (CBT) intervention program, including session topics, content focus, and delivery format.

Session	Title	Content	Homework
1	Relaxation	Introduction to relaxation strategies for pain management, including deep breathing, progressive muscle relaxation, and guided imagery	Daily practice of relaxation techniques (breathing, progressive muscle relaxation, guided imagery)
2	Chronic pain and cognitive behavioral therapy	Psychoeducation on chronic pain mechanisms and the cognitive behavioral therapy (CBT) model	Define personal goals to be achieved through the program
3	Case formulation; vicious cycle of chronic pain	Identification of cognitive, emotional, and behavioral factors maintaining chronic pain	Complete an individual case formulation related to pain maintenance
4	Cognitive flexibility training	Strategies to enhance cognitive flexibility by shifting perspectives and reducing rigid pain-related thinking	Practice attention-shifting exercises and record observations
5	Safety behavior analysis and behavioral activation	Identification of safety behaviors maintaining anxiety and avoidance, and introduction of behavioral activation techniques with pacing	Plan behavioral activation activities while considering pacing
6	Understanding and coping with neurodevelopmental traits	Identification of neurodevelopmental traits related to pain and development of individualized coping strategies	Identify and record personal neurodevelopmental traits
7	Attention-shifting training	Practice of attention-shifting exercises to improve focus and reduce pain-related attentional bias	Practice attention-shifting exercises and record observations
8	Monitoring of emotions and pain	Introduction of self-monitoring techniques for tracking emotions and pain levels, including the “three good things” exercise	Complete the “three good things” exercise daily
9	Assertiveness training	Identification of difficulties in pain-related communication and development of lower-stress coping responses	Summarize situations in which pain communication was unsuccessful
10	Anger management and cognitive restructuring	Training in anger management strategies and application of cognitive restructuring to challenge maladaptive thoughts	Complete a cognitive restructuring worksheet

Note. CBT-Th.F = therapist-guided cognitive behavioral therapy with follow-up support; CBT-Self = fully self-guided cognitive behavioral therapy; Sham = control condition involving daily submission of the day of the week and local weather.

#### 2.2.1 Guided digital CBT with therapist follow-up (CBT-Th.F)

Participants in this group received the digital CBT program delivered via LINE on their smartphone or tablet. Each 15-min video was provided daily. Participants watched the videos for five consecutive days, followed by a 30-min therapist-led follow-up session conducted online. Afterward, participants watched the remaining videos for another five days and completed a final follow-up session with the same therapist. Participants submitted homework via LINE, which was reviewed during therapist-led follow-up sessions. No formal therapist manual was used, because the follow-up sessions were intended only to check participants' progress and not to deliver therapeutic content. The two 30-min sessions were brief, semi-structured progress checks following a fixed agenda: participants' impressions of the videos, questions about the content and review of the homework submitted. Sessions were conducted by six clinical psychologists or certified public psychologists with 1–20 years of clinical experience.

#### 2.2.2 Self-guided digital CBT without therapist support (CBT-Self)

Participants in this group received the same digital CBT program as the CBT-Th.F group but did not receive any therapist follow-up sessions. All content was completed independently.

#### 2.2.3 Control condition (sham app)

Participants in the control group accessed LINE daily and submitted the day of the week and local weather conditions as a minimal engagement task. After completion of the intervention period, participants were offered access to the experimental program for two weeks on a voluntary basis. The sham condition was designed as a minimal attention/engagement control that matched the CBT arms in delivery channel (LINE), daily contact and duration, while containing no pain-related or therapeutic content. A pain-related task (e.g., a pain diary or reading about pain) was deliberately avoided, because self-monitoring and pain education can themselves have therapeutic effects.

### 2.3 Outcomes and measures

As this was a pilot feasibility trial, the primary outcomes were feasibility and pain catastrophizing. Pain intensity and interference (BPI) and well-being (WHO-5) were secondary outcomes; depressive (PHQ-9) and anxiety (GAD-7) symptoms were exploratory outcomes because they were not registered. Open-ended responses to the post-intervention questionnaire were summarized descriptively by one of the study therapists (R.T.) and reviewed by the first author (K.T.).

#### 2.3.1 Feasibility outcomes measure

Feasibility outcomes were recruitment (number screened and randomized), retention (completion of the post-intervention assessment), intervention completion and adherence (videos viewed and homework assignments submitted), attendance at therapist sessions (CBT-Th.F only), acceptability (post-intervention ratings of safety and perceived change, and open-ended feedback) and adverse effects. No progression criteria for a definitive trial were pre-specified.

#### 2.3.2 Primary outcome measure

##### 2.3.2.1 Pain-related cognition

Cognition about pain was assessed using the Pain Catastrophizing Scale (PCS). The PCS has demonstrated good internal consistency (Cronbach's α range: 0.67–0.87) (Sullivan et al., 1995). It consists of 13 items assessing catastrophic thinking related to pain, rated on a 5-point Likert scale ranging from 0 (“not at all”) to 4 (“all the time”). Total scores range from 0 to 52, with higher scores indicating greater pain catastrophizing. A score ≥ 30 has been proposed as a clinical cutoff.

#### 2.3.3 Secondary and exploratory outcome measures

##### 2.3.3.1 Comprehensive pain

Comprehensive pain and related outcomes were assessed using the Japanese version of the Brief Pain Inventory (BPI) (Uki et al., 1998), including total score, pain severity, and pain interference subscales. Items are rated on an 11-point numerical scale, with higher scores indicating greater pain severity or interference. The scale has demonstrated high reliability (Cronbach's α >0.80) and established validity.

##### 2.3.3.2 Mental well-being

Mental well-being was assessed using the World Health Organization Five Well-Being Index (WHO-5) (Topp et al., 2015). The WHO-5 consists of five items assessing subjective well-being over the past two weeks, with total scores ranging from 0 to 25. Scores below 13 suggest poor well-being and the need for further clinical assessment.

##### 2.3.3.3 Emotional distress. (exploratory outcomes)

Depressive symptoms were assessed using the Patient Health Questionnaire—9 (PHQ-9). The PHQ-9 has demonstrated diagnostic validity, with an overall accuracy of 85%, sensitivity of 75%, and specificity of 90% (Spitzer et al., 1999). It consists of nine items rated on a 4-point Likert scale, yielding total scores ranging from 0 to 27. Scores of 10 or higher indicate clinically significant depressive symptoms.

Anxiety symptoms were assessed using the Generalized Anxiety Disorder–7 (GAD-7) scale (Spitzer et al., 2006). The GAD-7 has demonstrated good reliability and validity, with a cutoff score of 10 optimizing sensitivity (89%) and specificity (82%). The scale consists of seven items rated on a 4-point Likert scale, with total scores ranging from 0 to 21.

#### 2.3.4 Baseline description measures

Baseline demographic characteristics included age, sex, education level, employment status, medical comorbidities, frequency of hospital visits, pain location, pain duration, and current prescriptions. Clinical baseline characteristics were assessed using the Somatic Symptom Disorder—B Criteria Scale (SSD-12; 12 items, total score 0–48) (Tominaga et al., 2025; Toussaint et al., 2018), pain intensity assessed with a numerical rating scale (NRS; 0–10) (Williamson and Hoggart, 2005), the Adult ADHD Self-Report Scale v1.1 (ASRS-v1.1; 18 items, total score 0–72) (Takeda et al., 2017) and the Autism-Spectrum Quotient (AQ; 50 items, total score 0–50) (Kurita et al., 2005), all of which have shown adequate reliability and validity in Japanese samples. To characterize the sample, a positive ASRS-v1.1 screen was defined as at least four of the six Part A items endorsed at or above the threshold frequency (Takeda et al., 2017), and the cut-off for the AQ was set at 26 (Kurita et al., 2005). Baseline characteristics are summarized in Table 2.

**Table 2:** Baseline demographic and clinical characteristics of participants across the three study groups.

	Treatment group		Control group	Total (n = 41)
	CBT-Th.F (n = 15)	CBT-Self (n = 15)	Sham (n = 11)	
Demographics				
Age, mean (SD)	31.1(3.31)	29.5(3.85)	26.8(3.74)	29.4(3.93)
Gender, n(%)				
Woman	9(60.0)	10(66.7)	6(54.5)	25(61.0)
Man	6(40.0)	5(33.3)	5(45.5)	16(39.0)
Education, n(%)				
High school or less	3(20.0)	4(26.7)	5(45.5)	12(29.3)
Some college/technical	2(13.3)	1(6.7)	1(9.1)	4(9.8)
Bachelor's degree or higher	10(66.7)	10(66.7)	5(45.5)	25(61.0)
Other	0(0)	0(0)	0(0)	0(0)
Employment, n(%)				
Fulltime work	10(66.7)	6(40.0)	4(36.4)	20(48.8)
Part-time work	4(26.7)	7(46.7)	3(27.3)	14(34.1)
Not working	1(6.7)	2(13.3)	4(36.4)	7(17.1)
Comorbidities, n(%)				
Physical symptoms	6(40.0)	7(46.7)	6(54.5)	19(46.3)
Psychiatric symptoms	10(66.7)	12(80.0)	7(63.6)	29(70.7)
Rate of visiting a hospital, n(%)				
Physical medicine	5(33.3)	7(46.7)	4(36.4)	16(39.0)
Mental medicine	10(66.7)	12(80.0)	7(63.6)	29(70.7)
Location of pain, n(%)				
Head	5(33.3)	5(33.3)	4(36.4)	14(34.1)
Neck	1(6.7)	0(0)	0(0)	1(2.4)
Back	0(0)	3(20.0)	0(0)	3(7.3)
Shoulder	5(33.3)	1(6.7)	0(0)	6(14.6)
Arm	0(0)	0(0)	1(9.1)	1(2.4)
Waist	1(6.7)	1(6.7)	3(27.3)	5(12.2)
Leg	1(6.7)	1(6.7)	0(0)	2(4.9)
Whole body/other	2(13.3)	4(26.7)	3(27.3)	9(22.0)
Duration of pain, n(%)				
<1 year	0(0)	1(6.7)	0(0)	1(2.4)
1–5 year	6(40.0)	4(26.7)	4(36.4)	14(34.1)
6–10 year	3(20.0)	8(53.3)	3(27.3)	14(34.1)
11–15 year	1(6.7)	1(6.7)	0(0)	2(4.9)
≥16 years	5(33.3)	1(6.7)	4(36.4)	10(24.4)
Prescription painkillers	8(53.3)	4(26.7)	4(36.4)	16(39.0)
Medication for NDD	5(33.3)	2(13.3)	2(18.2)	9(22.0)
Clinical characteristics				
SSD-12, mean(SD)	32.7(7.50)	33.8(10.00)	29.7(8.83)	32.3(8.8)
NRS, mean(SD)	6.87(1.19)	6.94(1.24)	7.00(1.01)	6.93(1.16)
ASRS, mean(SD)	36.5(17.2)	40.3(15.4)	34.3(17.3)	37.3(16.4)
ASRS Part A screen positive, n(%)	8(53.3)	11(73.3)	6(54.5)	25(61.0)
AQ, mean(SD)	28.3(7.1)	30.7(7.2)	28.7(8.8)	29.3(7.5)
AQ ≥ 26, n(%)	8(53.3)	11(73.3)	8(72.7)	27(65.9)

Note. Baseline data were not obtained for three randomized participants (CBT-Self, n = 1; sham, n = 2) due to loss of contact immediately after allocation. Values are presented as mean (SD) or n (%), unless otherwise indicated. Percentages may not sum to 100 because of rounding. ASD and ADHD diagnoses were self-reported. SSD-12 = Somatic Symptom Disorder—B Criteria Scale; NRS = Numerical Rating Scale; ASRS = Adult ADHD Self-Report Scale v1.1 (total score 0–72); ASRS Part A screen positive = four or more of the six Part A items at or above the threshold (items 1–3: “sometimes” or more often; items 4–6: “often” or more often); AQ = Autism-Spectrum Quotient (cut-off ≥26 for the Japanese version); NDD = neurodevelopmental disorder.

#### 2.3.5 Adverse event

Adverse events were monitored passively: participants were asked to report any adverse events to the research team by email. In the event of a serious adverse event, the research team was prepared to consult the physician in charge promptly.

### 2.4 Statistical analysis

#### 2.4.1 Quantitative analysis (primary and secondary outcomes)

No formal sample size calculation was conducted; a target of approximately 12–15 participants per group was set pragmatically for this pilot feasibility trial, and the trial was not powered to detect between-group differences. Feasibility outcomes were summarized descriptively.

Three participants (CBT-Self 1, sham 2) provided no outcome data, and one participant (CBT-Th.F) discontinued after five of ten sessions and provided baseline data only. The primary analysis was a mixed model for repeated measures (MMRM) including all participants with at least one observation (n = 41), with fixed effects of group, time and the group × time interaction, an unstructured covariance matrix, restricted maximum likelihood estimation and Satterthwaite degrees of freedom; this approach provides valid inference under the missing-at-random assumption and was consistent with the analysis plan approved by the ethics committee. Pairwise contrasts of change between groups (CBT-Self vs sham, CBT-Th.F vs sham and CBT-Self vs CBT-Th.F) followed Fisher's protected least significant difference procedure. As a sensitivity analysis, post-intervention scores were compared using analysis of covariance (ANCOVA) adjusted for baseline scores in participants with complete data (n = 40). Effect sizes for between-group differences in change were calculated as Hedges' g from change scores in participants with complete data. All between-group estimates are reported with 95% confidence intervals and regarded as preliminary. All tests were two-sided with a significance level of 0.05. Analyses were conducted using IBM SPSS Statistics ver. 32.0.0.0 (Advanced Statistics module; IBM Corp.). Given the pilot nature of the trial, findings were interpreted primarily in terms of effect sizes and uncertainty rather than definitive hypothesis testing.

### 2.5 Ethics and trial registration

The study protocol was approved by the Institutional Review Board of the Faculty of Medicine, Chiba University (No. M10731). The trial was registered with the University Hospital Medical Information Network (UMIN) Clinical Trials Registry (UMIN000056363). The UMIN registry does not include a field for the statistical analysis plan; the analysis plan was specified in the protocol approved by the ethics committee.

## 3 Results

### 3.1 Participants descriptive

Of 2416 panel members assessed for eligibility, 82 were screened and 44 were randomized to CBT-Th.F (n = 15), CBT-Self (n = 16) or sham (n = 13) (Fig. 1). Three participants (CBT-Self, n = 1; sham, n = 2) could not be contacted after allocation and provided no baseline or post-intervention data. One participant in the CBT-Th.F group discontinued the intervention after five of ten sessions, reporting a temporary physical health problem, and did not complete the post-intervention assessment. Thus, 41 participants (CBT-Th.F, n = 15; CBT-Self, n = 15; sham, n = 11) provided baseline data and were included in the MMRM, and 40 (CBT-Th.F, n = 14; CBT-Self, n = 15; sham, n = 11) provided data at both time points and were included in the complete-case analyses (ANCOVA and Hedges' g).

**Fig. 1:**
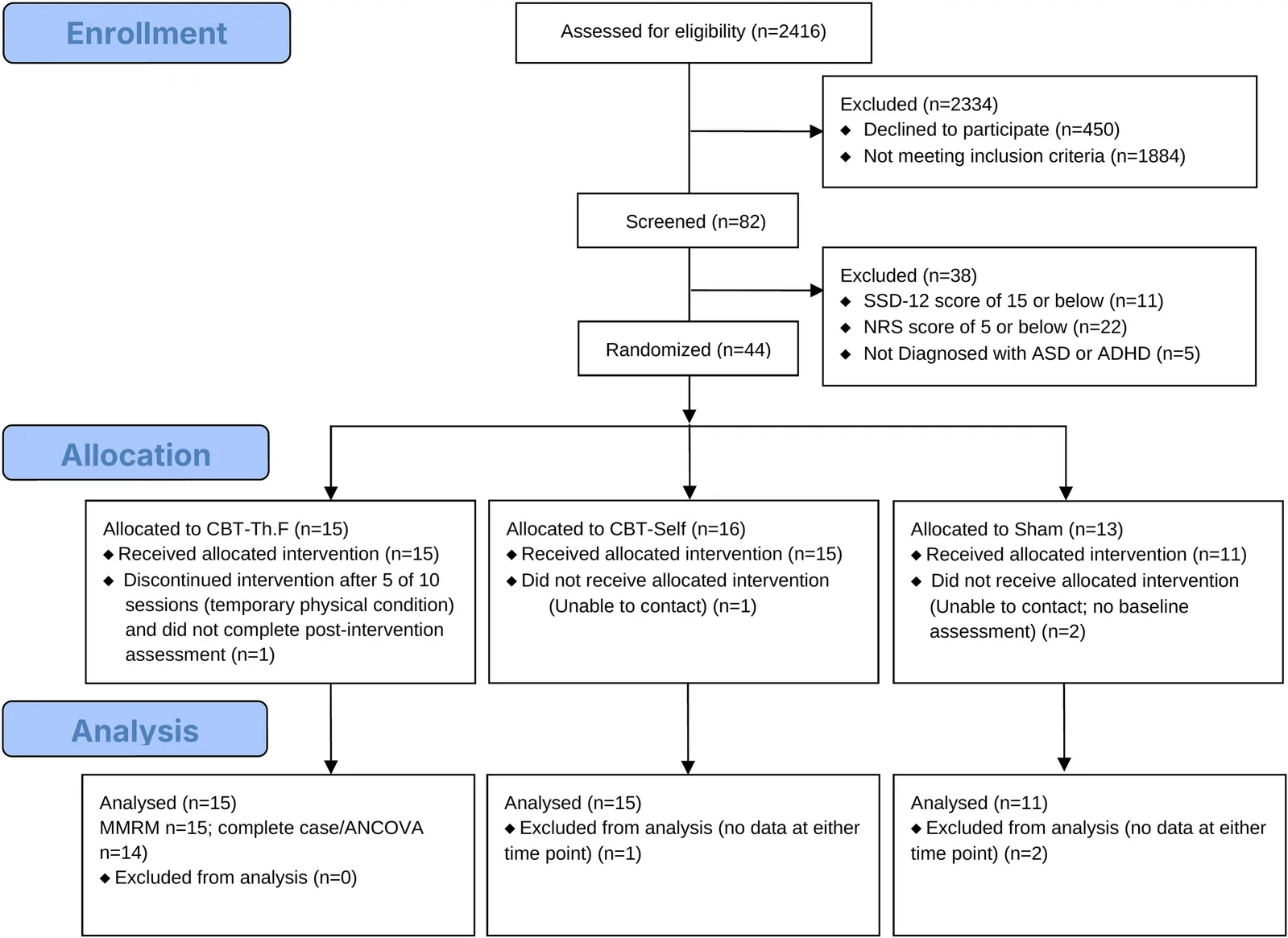
CONSORT flow diagram of participant recruitment, randomization, allocation and inclusion in the analyses.

Baseline characteristics are shown in Table 2. The mean age was 29.4 years (SD = 3.93), and 25 of 41 participants (61.0%) were women. Twenty-seven participants (65.9%) scored at or above the AQ cut-off of 26, and 25 (61.0%) screened positive on the ASRS-v1.1 Part A.

### 3.2 Feasibility, adherence and acceptability

Feasibility outcomes are summarized in Table 3. Of the 82 participants screened during the two-week recruitment period, 44 (53.7%) were randomized. The post-intervention assessment was completed by 40 of 44 randomized participants (90.9%): 14 of 15 (93.3%) in CBT-Th.F, 15 of 16 (93.8%) in CBT-Self and 11 of 13 (84.6%) in sham. Of the 31 participants allocated to the CBT arms, 30 started the program and 29 completed it (93.5% of those allocated; 96.7% of those who started). All participants who did not discontinue viewed all ten videos (mean 9.7 in CBT-Th.F and 10.0 in CBT-Self). Homework submission was lower than video viewing: participants submitted a mean of 6.3 of 10 assignments (range 0–10) in CBT-Th.F and 3.1 (range 0–9) in CBT-Self, and 11 of 15 (73.3%) and 7 of 15 (46.7%), respectively, submitted at least one assignment. In CBT-Th.F, 14 of 15 participants (93.3%) attended all scheduled therapist sessions. All 11 participants in the sham group who started the condition completed all daily submissions.

**Table 3:** Feasibility, adherence, acceptability and safety outcomes.

	CBT-Th.F	CBT-Self	Sham	Total
**Recruitment**				
Screened, n				82
Randomized, n (% of screened)	15	16	13	44 (53.7)
**Retention**				
Baseline assessment completed, n/N (%)	15/15 (100)	15/16 (93.8)	11/13 (84.6)	41/44 (93.2)
Post-intervention assessment completed, n/N (%)	14/15 (93.3)	15/16 (93.8)	11/13 (84.6)	40/44 (90.9)
**Intervention completion and adherence**				
Started allocated intervention, n/N (%)	15/15 (100)	15/16 (93.8)	11/13 (84.6)	41/44 (93.2)
Completed allocated intervention, n/N started (%)	14/15 (93.3)	15/15 (100)	–	29/30 (96.7)
Videos viewed (of 10), mean (range)^a^	9.7 (5–10)	10.0 (10−10)	–	9.8 (5–10) b
Homework assignments submitted (of 10), mean (range)	6.3 (0−10)	3.1 (0–9)	–	4.7 (0–10) b
Submitted at least one homework assignment, n/N (%)	11/15 (73.3)	7/15 (46.7)	–	18/30 (60.0)^b^
Completed all daily submissions (sham), n/N (%)	–	–	11/11 (100)	–
Attended all scheduled therapist sessions, n/N (%)	14/15 (93.3)	–	–	–
**Acceptability**^c^				
Rated the program as safe to use, n/N (%)	14/14 (100)	15/15 (100)	NA	29/29 (100)^b^
Self-rated change: very improved/slightly improved/no change/very much worsened, n	1/8/4/1	1/9/5/0	NA	2/17/9/1
**Safety**				
Adverse events reported spontaneously (email), n	0	0	0	0
Discontinued intervention because of a health problem, n	1^d^	0	0	1

Note. NA = not administered.

a Number of the ten daily videos viewed among participants who started the program; all participants who did not discontinue viewed all ten videos.

b CBT arms only (n = 30 started; n = 29 for post-intervention items).

c Post-intervention questionnaire; the safety item was not administered in the sham arm.

d One participant withdrew after five of ten sessions, reporting a temporary physical health problem; no further information was available and its relationship to the intervention could not be assessed.

All 29 respondents in the CBT arms rated the program as safe to use. In self-rated change after the intervention, 2 respondents (6.9%) reported being “very improved”, 17 (58.6%) “slightly improved”, 9 (31.0%) “no change” and 1 (3.4%) “very much worsened” (Table 3).

In open-ended responses to the post-intervention questionnaire, participants most often mentioned a better understanding of the relationship between pain and neurodevelopmental traits, greater awareness of pain-related thinking patterns and attentional processes, and the application of learned strategies in daily life. Components described as helpful included psychoeducation on neurodevelopmental traits, attention shifting, assertiveness training, safety behavior analysis and behavioral activation. Suggestions for improvement included brief downloadable written summaries of the video content and more consistent audio quality and volume. Because these responses were summarized by members of the research team, they are presented descriptively.

### 3.3 Primary outcome; pain catastrophizing

Results for all outcomes are presented in Table 4. Among participants with complete data, the mean baseline PCS score was higher in the CBT-Self group (38.20) than in the CBT-Th.F (30.64) and sham (30.91) groups. For the PCS, the MMRM showed a significant Group × Time interaction (F(2, 37.3) = 4.45, *p* = .018). PCS scores decreased from baseline to post-intervention in CBT-Th.F (from 30.64 to 24.86; mean change −5.79, SD = 8.81) and CBT-Self (from 38.20 to 29.73; mean change −8.47, SD = 9.48), and increased in sham (from 30.91 to 33.45; mean change +2.55, SD = 10.50). Compared with sham, the estimated difference in change was −11.01 points (95% CI −18.68 to −3.35; *p* = .006; Hedges' g = −1.08) for CBT-Self and −8.41 points (95% CI −16.18 to −0.65; *p* = .035; g = −0.84) for CBT-Th.F. In the direct comparison between the two CBT formats, the difference in change (CBT-Self minus CBT-Th.F) was −2.60 points (95% CI −9.76 to 4.56; *p* = .467; g = −0.28).

**Table 4:** Pre-and post-intervention scores and between-group estimates for primary, secondary and exploratory outcomes.

Outcome	Group	Baseline (T0)		Post (T1)		Change (T1 − T0)		Group × Time (MMRM)		Difference in change (MMRM)		Hedges' g	ANCOVA (sensitivity)	
		Mean	SD	Mean	SD	Mean	SD	F	*p*	Estimate [95% CI]	*p*		Adjusted difference [95% CI]	*p*
PCS	CBT-Th.F (n = 14)	30.64	10.65	24.86	10.82	−5.79	8.81	4.45	0.018	−8.41 [−16.18, −0.65]	0.035	−0.84	−8.41 [−15.89, −0.92]	0.029
	CBT-Self (n = 15)	38.20	7.89	29.73	10.98	−8.47	9.48			−11.01 [−18.68, −3.35]	0.006	−1.08	−8.99 [−16.64, −1.35]	0.022
	Sham (n = 11)	30.91	14.76	33.45	14.75	2.55	10.50							
	CBT-Self vs CBT-Th.F									−2.60 [−9.76, 4.56]	0.467	−0.28	−0.59 [−7.80, 6.63]	0.870
BPI total	CBT-Th.F (n = 14)	3.33	2.60	2.64	1.72	−0.68	3.02	0.02	0.977	−0.16 [−2.17, 1.85]	0.871	−0.06	–	
	CBT-Self (n = 15)	3.57	2.46	3.08	2.01	−0.49	2.25			0.02 [−1.98, 2.01]	0.985	0.01	–	
	Sham (n = 11)	4.13	2.39	3.62	2.33	−0.51	2.03							
	CBT-Self vs CBT-Th.F									0.18 [−1.67, 2.03]	0.843	0.07	–	
BPI severity	CBT-Th.F (n = 14)	3.68	2.58	2.98	1.77	−0.70	2.99	0.06	0.938	−0.18 [−2.27, 1.92]	0.867	−0.07	–	
	CBT-Self (n = 15)	3.55	2.16	3.22	2.05	−0.33	2.48			0.17 [−1.91, 2.25]	0.872	0.07	–	
	Sham (n = 11)	3.75	2.08	3.25	2.22	−0.50	2.21							
	CBT-Self vs CBT-Th.F									0.34 [−1.59, 2.27]	0.723	0.13	–	
BPI interference	CBT-Th.F (n = 14)	2.98	2.85	2.31	1.77	−0.67	3.23	0.01	0.989	−0.15 [−2.30, 2.00]	0.889	−0.05	–	
	CBT-Self (n = 15)	3.58	3.06	2.93	2.11	−0.65	2.50			−0.13 [−2.26, 2.01]	0.904	−0.05	–	
	Sham (n = 11)	4.51	2.81	3.99	2.62	−0.52	2.01							
	CBT-Self vs CBT-Th.F									0.02 [−1.96, 2.00]	0.983	0.01	–	
WHO-5	CBT-Th.F (n = 14)	12.07	6.94	12.21	6.53	0.14	3.90	1.99	0.151	3.64 [−0.87, 8.14]	0.111	0.59	–	
	CBT-Self (n = 15)	10.00	5.42	10.67	4.39	0.67	4.98			4.12 [−0.34, 8.58]	0.069	0.63	–	
	Sham (n = 11)	11.00	6.18	7.55	5.15	−3.45	7.78							
	CBT-Self vs CBT-Th.F									0.49 [−3.67, 4.64]	0.814	0.11	–	
PHQ-9†	CBT-Th.F (n = 14)	14.50	6.35	11.71	6.74	−2.79	5.47	4.48	0.018	−6.64 [−11.91, −1.38]	0.015	−0.92	−7.46 [−12.05, −2.87]	0.002
	CBT-Self (n = 15)	15.53	5.62	12.40	6.64	−3.13	5.51			−7.04 [−12.25, −1.84]	0.009	−0.97	−7.28 [−11.78, −2.78]	0.002
	Sham (n = 11)	16.00	8.50	19.91	5.68	3.91	8.65							
	CBT-Self vs CBT-Th.F									−0.40 [−5.25, 4.45]	0.869	−0.06	0.18 [−4.04, 4.40]	0.932
GAD-7†	CBT-Th.F (n = 14)	10.57	4.16	8.86	4.87	−1.71	5.14	0.43	0.653	−1.63 [−5.25, 1.99]	0.368	−0.37	–	
	CBT-Self (n = 15)	10.67	6.15	9.40	5.88	−1.27	4.73			−1.18 [−4.75, 2.40]	0.510	−0.28	–	
	Sham (n = 11)	11.27	7.59	11.18	7.33	−0.09	2.81							
	CBT-Self vs CBT-Th.F									0.46 [−2.88, 3.80]	0.784	0.09	–	

Note. Means and SDs are based on participants with data at both time points (n = 40). MMRM estimates include all participants with at least one assessment (n = 41). Difference in change = change in the first-named group minus change in the comparison group (sham unless otherwise indicated); negative values favor the first-named group for all outcomes except the WHO-5, for which positive values favor the first-named group. Hedges' g was calculated from change scores. ANCOVA (post-intervention score adjusted for baseline) was performed only for outcomes with a significant Group × Time interaction. PCS, Pain Catastrophizing Scale; BPI, Brief Pain Inventory; WHO-5, World Health Organization—Five Well-Being Index; PHQ-9, Patient Health Questionnaire-9; GAD-7, Generalized Anxiety Disorder-7; MMRM, mixed model for repeated measures; ANCOVA, analysis of covariance; CI, confidence interval.

† Exploratory outcomes (not registered in UMIN-CTR).

The baseline-adjusted ANCOVA (sensitivity analysis) gave similar results (group effect F(2, 36) = 3.52, *p* = .040, partial η^2^ = 0.16): the adjusted difference was −8.99 points (95% CI −16.64 to −1.35; *p* = .022) for CBT-Self versus sham and −8.41 points (95% CI −15.89 to −0.92; *p* = .029) for CBT-Th.F versus sham. After adjustment for baseline, the difference between the two CBT formats decreased from −2.60 to −0.59 points (95% CI −7.80 to 6.63; *p* = .870). All confidence intervals were wide.

### 3.4 Secondary outcomes: pain intensity, interference and well-being

No Group × Time interaction was found for the BPI total score (F = 0.02, *p* = .977), pain severity (F = 0.06, *p* = .938) or pain interference (F = 0.01, *p* = .989). BPI scores decreased slightly in all three groups (mean changes from −0.33 to −0.70 points). For the WHO-5, the interaction was not significant (F = 1.99, *p* = .151); scores changed little in the CBT arms (+0.14 in CBT-Th.F and +0.67 in CBT-Self) and decreased in sham (−3.45). In line with the analysis plan, pairwise contrasts for these outcomes were not interpreted; the estimates are reported in Table 4 for completeness.

### 3.5 Exploratory outcomes: depressive and anxiety symptoms

For the PHQ-9, the MMRM showed a significant Group × Time interaction (F(2, 37.9) = 4.48, *p* = .018). Scores decreased in CBT-Th.F (mean change −2.79) and CBT-Self (−3.13) and increased in sham (+3.91). Compared with sham, the estimated difference in change was −6.64 points (95% CI −11.91 to −1.38; *p* = .015) for CBT-Th.F and −7.04 points (95% CI −12.25 to −1.84; *p* = .009) for CBT-Self; the difference between the two CBT formats was −0.40 points (95% CI −5.25 to 4.45; *p* = .869). The ANCOVA gave similar estimates (group effect F(2, 36) = 6.90, *p* = .003, partial η^2^ = 0.28). The increase in the sham group was concentrated in two participants, whose scores increased by 11 and 26 points. For the GAD-7, no Group × Time interaction was found (F = 0.43, *p* = .653).

### 3.6 Adverse events and negative effects

No adverse events were reported spontaneously by email in any group. One participant in CBT-Th.F discontinued the intervention after five sessions, reporting a temporary physical health problem; no further information was available, and its relationship to the intervention could not be assessed. One respondent in CBT-Th.F rated themselves as “very much worsened” after the intervention; the same participant rated the program as safe to use.

## 4 Discussion

Although NDD traits are common among patients with intractable chronic pain (Kasahara et al., 2026; Miyoshi et al., 2025), few interventions for chronic pain have taken these traits into account (Garriga-Cazorla et al., 2025). To our knowledge, this pilot trial is the first to examine the feasibility and acceptability of a low-intensity digital CBT-based psychoeducational program tailored to adults with chronic pain and NDD traits, delivered in therapist-supported and self-guided formats, and to obtain preliminary estimates of change in pain catastrophizing. Retention was high (90.9% completed the post-intervention assessment), 29 of the 30 participants who started the program completed it (96.7%), 65.5% of respondents rated themselves as improved, and all respondents rated the program as safe to use. Pain catastrophizing decreased more in both CBT formats than in the sham condition, but the estimates were imprecise, and no difference between the two formats was found.

The program was deliberately limited to two weeks of brief daily videos. Adherence to self-guided internet interventions declines as programs become longer (Christensen et al., 2009; Christensen et al., 2006), and everyday distractions and difficulty resuming after breaks are common reasons for dropout (Simon et al., 2025). These difficulties may be amplified by NDD traits, such as distractibility and forgetfulness in ADHD (Nordby et al., 2022) and difficulty initiating activities and the need for frequent reminders in ASD (Westerberg et al., 2023), in addition to the burden of chronic pain. In unguided digital self-management interventions for chronic pain, system use typically declines over time; for example, use of a 12-week smartphone program fell below 50% after week 5 (MacLean et al., 2025). Moreover, participants who were behind schedule in the second week of an internet-based CBT program finished fewer treatment modules (Gasslander et al., 2021). A two-week program may therefore fit within the period in which engagement can be sustained.

Consistent with this rationale, nearly all participants viewed all daily videos. Program completion (96.7%) exceeded the range reported in a systematic review of unguided digital self-management interventions for chronic pain, in which 19% to 81% of participants completed all intervention components (MacLean et al., 2025), and retention and completion also compared favorably with the 70% retention and 46% completion of an unguided 8-week web-based self-management program (Marier-Deschenes et al., 2024) and the 80% completion of an internet-Based Cognitive-Behavioral Program delivered as five lessons over 8 weeks with clinician support (Navin et al., 2026). However, cross-study comparisons should be made cautiously and payment through the research panel may have contributed to retention (Linardon and Fuller-Tyszkiewicz, 2020). In contrast, homework submission, an indicator of skill practice that is rarely reported in digital pain self-management studies (MacLean et al., 2025), was modest: participants submitted a mean of 6.3 of 10 assignments in CBT-Th.F and 3.1 in CBT-Self, and 4 and 8 participants, respectively, submitted none. Thus, the brief daily format appeared sufficient to sustain viewing of the videos, but not active practice, which is a core component of CBT. The descriptively higher submission in CBT-Th.F suggests that even brief contact with a therapist may support practice.

Compared with sham, both CBT formats showed greater reductions in pain catastrophizing and, as an exploratory outcome, in depressive symptoms. These estimates should be interpreted cautiously. First, the mean within-group reductions in PCS (−5.79 in CBT-Th.F and −8.47 in CBT-Self) were smaller than the minimal important change of 11 points reported for patients with PCS scores above 30 (Monticone et al., 2021). Second, the between-group differences partly reflected deterioration in the sham group (PCS +2.55; PHQ-9 +3.91), and the increase in PHQ-9 was concentrated in two participants. Possible explanations include the burden of a daily task without therapeutic content and disappointment at allocation to the control arm. Because the sham condition did not resemble a treatment and treatment credibility and expectancy were not measured, expectancy effects in the CBT arms cannot be ruled out. In addition, whether the PHQ-9 measured depressive symptoms equivalently across groups and time points could not be examined (Hlynsson et al., 2025). Third, there was no evidence of a difference between the two CBT formats; the larger raw change in CBT-Self may be explained by chance and by its higher baseline PCS score (regression to the mean). Finally, no between-group differences were found for pain intensity, pain interference or well-being. Taken together, these findings suggest that two weeks of brief psychoeducational videos may change pain-related cognition in the short term but may be insufficient as a standalone treatment.

Several limitations should be considered when interpreting the present findings. First, the sample was small, and the trial was not powered to detect between-group differences; therefore, no conclusions about efficacy can be drawn. Second, no follow-up assessment was conducted, and the intervention period was short; it therefore remains unclear whether any changes were sustained. Third, the generalizability of the findings is limited: participants were recruited from a commercial online research panel, their mean age was relatively young (29.4 years), and ASD and ADHD diagnoses were based on a single self-reported item that did not distinguish between the two. Fourth, adverse events were monitored only through spontaneous reports by email, so negative effects may have been under-detected.

Although preliminary, these findings suggest several directions for developing the program. First, several videos already included brief guided practice (e.g., relaxation and attention-shifting exercises), and homework asked participants to continue this practice in daily life. Homework submission was nevertheless low, particularly in CBT-Self; this is unlikely to be explained by program length alone, because forgetting, everyday distractions and low motivation are common reasons for non-completion of self-guided programs (Nordby et al., 2022; Simon et al., 2025). Submission was descriptively higher in CBT-Th.F, in which submitted homework was reviewed in the follow-up sessions, although changes in outcomes did not differ between the two formats. In this format, however, feedback was given only twice, after five and ten days. Rather than extending the program, a future version might therefore combine the in-video practice with brief daily prompts to practice, a simple daily record of practice, and immediate feedback on the recorded practice to reinforce it. In a trial of an internet-delivered pain management program, outcomes did not differ consistently according to the amount of clinician contact (Dear et al., 2015), and in social anxiety disorder, the short-term advantage of guidance disappeared at follow-up (Mechler et al., 2024). These findings suggest that the timing and content of support, rather than its amount, may need to be considered; for example, support could focus specifically on practice and be provided immediately after it is recorded. A definitive trial should be adequately powered, include follow-up assessments and verified diagnoses, measure treatment credibility and expectancy, which predict adherence (Gasslander et al., 2021), and monitor negative effects systematically, for example with the Negative Effects Questionnaire (Rozental et al., 2019). Whether brief psychoeducation on NDD traits and pain could serve as preparation for more intensive psychological treatment should also be examined.

## 5 Conclusion

This pilot trial suggests that a brief digital CBT program for adults with chronic pain and NDD traits is feasible and acceptable. Preliminary estimates for pain catastrophizing favored both CBT formats over a sham condition but were imprecise, and no difference between the therapist-supported and self-guided formats was detected. Before a definitive trial, the program should be refined to better support practice of the skills it teaches.

## Declaration of Generative AI and AI-assisted technologies in the writing process

During the preparation of this work, the author used Claude (Anthropic) and ChatGPT (OpenAI) for language checking, proofreading, and brainstorming during the manuscript preparation process. After using these tools, the author reviewed and edited the content as needed and takes full responsibility for the content of the published article.

## Declaration of competing interest

The authors declare that they have no known competing financial interests or personal relationships that could have appeared to influence the work reported in this paper.
